# Endovascular Neuromodulation: Safety Profile and Future Directions

**DOI:** 10.3389/fneur.2020.00351

**Published:** 2020-04-24

**Authors:** Samad A. Raza, Nicholas L. Opie, Andrew Morokoff, Rahul P. Sharma, Peter J. Mitchell, Thomas J. Oxley

**Affiliations:** ^1^Department of Neurosurgery, Royal Melbourne Hospital, Melbourne, VIC, Australia; ^2^Department of Medicine, Vascular Bionics Laboratory, Melbourne Brain Centre, The University of Melbourne, Melbourne, VIC, Australia; ^3^Interventional Cardiology, Stanford Health Care, Palo Alto, CA, United States; ^4^Department of Radiology, The University of Melbourne & The Royal Melbourne Hospital, Melbourne, VIC, Australia; ^5^Departments of Medicine and Neurology, Melbourne Brain Centre at The Royal Melbourne Hospital, The University of Melbourne, Melbourne, VIC, Australia; ^6^Department of Neurosurgery, Mount Sinai Hospital, New York, NY, United States

**Keywords:** Stentrode^TM^, neuromodulation, endovascular, brain-machine interface, stent, lead, thrombosis

## Abstract

Endovascular neuromodulation is an emerging technology that represents a synthesis between interventional neurology and neural engineering. The prototypical endovascular neural interface is the Stentrode^TM^, a stent-electrode array which can be implanted into the superior sagittal sinus via percutaneous catheter venography, and transmits signals through a transvenous lead to a receiver located subcutaneously in the chest. Whilst the Stentrode^TM^ has been conceptually validated in ovine models, questions remain about the long term viability and safety of this device in human recipients. Although technical precedence for venous sinus stenting already exists in the setting of idiopathic intracranial hypertension, long term implantation of a lead within the intracranial veins has never been previously achieved. Contrastingly, transvenous leads have been successfully employed for decades in the setting of implantable cardiac pacemakers and defibrillators. In the current absence of human data on the Stentrode^TM^, the literature on these structurally comparable devices provides valuable lessons that can be translated to the setting of endovascular neuromodulation. This review will explore this literature in order to understand the potential risks of the Stentrode^TM^ and define avenues where further research and development are necessary in order to optimize this device for human application.

## Introduction

Intracranial neuromodulation has numerous potential applications, including epilepsy monitoring (1, 2), neurostimulation (3), thought to text and thought to speech paradigms (4–6), and control of robotic limbs and exoskeletons (7). A number of neural interfaces are in established clinical practice. These include electrocorticography (ECoG) and intraparenchymal depth electrodes (1), which enable intracranial recording of neural signals, and deep brain stimulation (DBS) (3), which enables therapeutic stimulation of deep brain nuclei. Since their implantation requires craniotomy, these devices carry the risks of open brain surgery, such as hemorrhage, infection, postoperative pain, and prolonged recovery time (8–11). Furthermore, surgically implanted electrodes induce chronic inflammation at the electrode-tissue interface, leading to changes in electrical impedance and signal degradation over time (12, 13). Endovascular neuromodulation may provide a minimally invasive solution that circumvents these issues whilst providing safe, accurate and long term recording, or stimulation of the brain (14, 15).

Given the proximity of cerebral vessels to numerous important brain regions (14, 16), the cerebrovascular system is a promising conduit for a neural interface. Most historical endovascular EEG devices have been limited to short term application ranging from hours to days (17–22). In contrast, the recently developed Stentrode^TM^, which is permanently implanted into the superior sagittal sinus (SSS) via percutaneous catheter venography, is capable of long-term application (14). In an ovine model, this device demonstrated stable endovascular EEG recordings of sensorimotor cortex for up to 190 days, with equivalent fidelity to extradural and subdural ECoG electrodes (14, 23), as well as stimulation of discrete neuronal populations in sheep motor cortex, with comparable somatotopic accuracy to surgically implanted depth electrodes (15).

Although a human trial of the Stentrode^TM^ is now underway (SWITCH trial - Stentrode^TM^ First in Human Early Feasibility Study), significant questions remain about its risk-benefit profile, should it progress to routine clinical application. Whilst the Stentrode^TM^'s endovascular delivery confers greater ease of implantation, it also relies on the transmission of signals through a permanent transvenous lead. Possible complications of this device include stent- or lead-associated venous thrombosis, device-related infection and lead failure (14). Given the current absence of human trial data on the Stentrode^TM^, existing literature on structurally comparable devices may provide valuable insights into these risks and their possible preventative strategies. For example, there is a growing number of studies on stent deployment in the intracranial sinuses for a range of indications (24–31), from which possible stent-related complications of the Stentrode^TM^ may be surmised. Although a transvenous lead has never been previously implanted in the human brain, substantial lessons about the safety and design characteristics of transvenous leads can be taken from the literature on cardiac electrotherapy devices (32–36). This review aims to translate these findings to the neurological setting and define avenues for further optimisation of the Stentrode^TM^ prototype.

## Ovine Studies of the Stentrode^TM^

For any novel biomedical device, rigorous animal testing is a necessary precursor to human trials. Whilst proof of concept for the Stentrode^TM^ currently only exists in ovine models (14, 15, 23, 37), these experiments provide important insights into the safety characteristics of the device.

Oxley et al. implanted the Stentrode^TM^ into the SSS of 20 sheep for up to 190 days (14). Whilst there was a slight reduction in lumen area of the SSS in animals implanted for over 100 days compared to animals implanted for less than seven days (38, 39), there were no cases of complete SSS occlusion. However, 37% of the cortical veins (3 out of a total of 8 veins in 3 animals) that entered the SSS at point of implantation were occluded at 3 months. It is noteworthy that none of the sheep with occluded cortical veins displayed neurological sequelae such as difficulty walking, reduced feeding or focal neurological signs (14). Hypothetically, this may be secondary to compensatory flow through collateral venous channels. For example, in human cohorts implanted with transvenous cardiac leads, it is known that collateral channels develop in order to reroute blood flow in response to lead-associated venous occlusion (40). Furthermore, there is some data to suggest that the presence and extent of collateral veins in the brain may influence the outcome in cerebral venous sinus thrombosis (41), lending support to this hypothesis. However, there was no post-implantation angiographic data presented in Oxley et al.'s study to confirm the formation of collateral channels. Such data would greatly contribute to our understanding of the long-term response of the cerebral vasculature to Stentrode^TM^ implantation.

The Stentrode^TM^ implantation procedure itself carries potential risks. In 39 sheep that underwent cerebral catheter venography (37) using 2, 4, 5, or 6 F coaxial catheters, significant catheter-related complications were encountered with catheters larger than 4 F. Thirty-three per cent, or 6 out of the 18 animals that underwent catheterisation with 5 F catheters, experienced subdural haematomas secondary to venous dissection. These dissections occurred at either the torcula herophili or the anterior SSS, and in 5 out of 6 cases resulted in non-recovery after anesthesia. Similarly, 6 F catheters had a 36% complication rate (4 out of 11 animals). Two of these complications were subdural hemorrhages secondary to torcula herophili dissection and both caused nonrecovery after anesthesia. The other two cases were thrombus formation within the SSS and were not associated with any clinically detectable sequelae. These may have been secondary to a longer procedure time associated with the 6 F catheter. Somewhat reassuringly, all catheterisations performed with the 2 and 4 F catheters were uncomplicated, suggesting that a small diameter catheter is favored for navigation into the anterior SSS for successful Stentrode^TM^ deployment (37).

There are some important remarks to be made about the generalizability of this ovine model to humans. The SSS in sheep was chosen as an initial target primarily because of its ease of access through catheter venography and its parallel orientation to the ovine motor area. Furthermore, it has a diameter of 1.2 to 2.4 mm (37), which is comparable to the diameter of the human CSV (2.3 to 4.9 mm) (14). The CSV is the ideal corresponding target in humans because it is adjacent and parallel to the coronally orientated primary motor cortex in the human brain. However, unlike the SSS, it lacks a dural wall which may have significant implications for stent safety and electrode incorporation into the vessel wall (37). Additionally, the clinical endpoints measured in the sheep model were limited to gross motor function. It was not possible to detect subtle cognitive deficits or speech disturbance in these animals. Furthermore, it is not possible to train sheep to perform complex motor tasks, making it difficult to assess the utility of the Stentrode^TM^ as a closed loop brain machine interface in an ovine model. These limitations highlight the necessity of a human trial of the Stentrode^TM^.

## Stent Safety: Lessons From Venous Sinus Stenting

There is an expanding body of literature on stenting of intracranial venous sinuses for a range of indications (24–31, 42–47). This literature exposes the possible risks of intracranial stenting, particularly in-stent thrombosis, and provides strategies for prevention of these complications, namely anticoagulation and antiplatelet therapy. The lessons from these studies may help to define optimum preventative strategies in human Stentrode^TM^ recipients.

Stenting of the SSS is not a new concept. Several case series and case reports describing SSS stenting exist (42–47) (summarized in Table 1). The largest clinical series of SSS stenting was performed by Raper et al. in 19 subjects with non-thrombotic veno-occlusive disease involving the SSS (42). Stents ranging in diameter from 8 to 10 mm were utilized. Either one, two or three stents were implanted adjacent to one another. Patients were premedicated with 7 days of aspirin and clopidogrel and received 100 units/kg of IV heparin during the procedure. Whilst two patients suffered a femoral artery pseudoaneurysm at the puncture site, there were no intraprocedural complications and no stent associated stenosis at a median follow-up of 5.2 months (42). Similarly, in all other case reports of SSS stenting, the SSS remained patent at long-term follow-up (43–47).

**Table 1:** Case series and case reports of SSS stenting.

**References**	***N***	**Indication**	**Stent model and dimensions**	**Implant location**	**Antithrombotic regimen**	**Outcome and complications**
Raper et al. (42)	19	Non-thrombotic veno-occlusive disease.	Stent models not specified. Stent diameters of 8 and 10 mm. Total length of stent constructs ranged from 60 to 180 mm.	One, two or three stents deployed in tandem in proximal SSS (posterior to vein of Trolard). In 12 patients the stent construct spanned into the traverse sinus. In 6 patients the stent construct spanned into the sigmoid sinus.	7 days of premedication with aspirin and clopidogrel. Intravenous heparin 100 units/kg during procedure.	No intraprocedural complications. Two patients developed femoral artery pseudoaneurysm at arterial puncture site. All stents remained patent and no patients had junctional SSS stenosis distal to the stent construct, at a mean follow-up of 5.2 months.
Matsumoto et al. (43)	1	CVST of SSS	ENTERPRISE Vascular Reconstruction Device (Codman & Shurtleff, Johnson & Johnson). Diameter 4.5 mm.	Three stents placed in tandem in posterior SSS	Intravenous heparin to achieve an APTT of 250-300 seconds. Postoperative anticoagulation for three months (agent not specified).	SSS patent at 3 months. No recurrence of CVST at 3 year follow-up.
Matsumoto et al. (44)	2	CVST of SSS	ENTERPRISE VRD. Diameter not specified	Two stents placed in tandem in posterior SSS.	Preoperative anticoagulation. aspirin 200 mg and clopidogrel 300 mg load on table. Anticoagulation and antiplatelet therapy continued postoperatively.	SSS patent at long-term follow-up (2 years in first patient and 1.5 years in second patient).
Ohara et al. (45)	1	Occlusion of posterior third of SSS in setting of dural arteriovenous fistulas.	Carotid WALLSTENT (Boston Scientific). Length 21 mm, diameter 8 mm.	Three stents placed in tandem in posterior SSS.	Intravenous heparin during procedure and aspirin loading dose on table. No postoperative antithrombotics.	SSS patent at 3 months follow-up
Entezami et al. (46)	1	SSS stenosis due to external compression by parasagittal meningioma	Carotid WALLSTENT Length 21 mm, diameter 8 mm.	Single stent in posterior third of SSS.	Seven days of 325 mg daily aspirin preoperatively, continued for 6 months postoperatively, followed by 81 mg daily aspirin indefinitely.	SSS patent at 3 months follow-up.
Ganesan et al. (47)	1	SSS stenosis due to external compression by parasagittal meningioma	Omnilink.018 balloon-mounted stent. Diameter not specified.	Single stent in posterior SSS.	Intravenous heparin during procedure. Warfarin postoperatively for 8 weeks, then indefinite aspirin.	SSS patent at 14 months follow-up.

*APTT, activated partial thromboplastin time; CVST, cerebral venous sinus thrombosis; SSS, superior sagittal sinus; VRD, vascular reconstruction device*.

Greater experience exists with stenting of the transverse sinuses for the treatment of idiopathic intracranial hypertension (24–31). According to recent systematic reviews, the overall complication rate associated with this intervention, including minor complications such as wound hematomas or pseudoaneurysms, is 1–7% (24–29). Major neurological complications, such as in-stent thrombosis and subdural, extradural, subarachnoid, or intracerebral hemorrhage, due to venous injury, occur at a rate of 1–3% (24–29). Delayed re-stenosis within, or adjacent to, the stent construct is another undesirable outcome after this therapy. According to one systematic review the rate of in-stent stenosis was 3.4% after a mean radiographic follow-up period of 15.2 months (28). Stent-adjacent stenosis is comparatively more common, occurring in approximately 10-15% of patients (28, 29). However, these complications may be secondary to high intracranial pressure gradients encountered in patients with IIH and may be less likely if stents were to be implanted in individuals with normal intracranial pressure. Additional factors that limit the generalizability of these studies to future Stentrode^TM^ recipients include their non-randomized retrospective design and significant inter-cohort heterogeneity.

It must be noted that there is currently minimal clinical experience with stenting of the anterior part of the SSS, which is the planned Stentrode^TM^ implantation site. The entry point of the central sulcal vein may pose a particular risk in this region, given that this vein drains the sensorimotor cortex (14). As an analogy, a similar threat is posed to the vein of Labbe (VOL) in transverse sinus stenting procedures. Interestingly, data from retrospective studies in this setting suggest that occlusion of the VOL is a rare event, even when the ostium of the vein is traversed by an implanted stent (30, 31). For example, in a series of 56 patients by Raper et al., the implanted stent crossed the VOL ostium in 92.9% of cases. Immediate postoperative angiograms were performed on 32 of these patients, showing that only 1 patient (3.1%) had complete VOL occlusion, whilst 6 patients (18.8%) had sluggish VOL filling and a further 1 patient had diminished VOL caliber with normal transit time. At 3 months follow-up, 5 out of 6 patients with initially sluggish filling improved to normal transit time, whilst 1 patient went on to have complete VOL occlusion. Importantly, there were no neurological sequelae in any of the patients with altered VOL drainage patterns (30). This correlates with the ovine data on Stentrode^TM^ implantation, in which sheep with bridging vein occlusion displayed no clinical deficits (14).

In-stent thrombosis is a potentially serious complication that could result in adverse neurological outcomes in Stentrode^TM^ recipients. The risk of in-stent thrombosis can be minimized through the use of antiplatelets and anticoagulants in the perioperative period (26). However, the ideal antithrombotic regimen for intracranial venous sinus stenting is not well defined. A systematic review of transverse sinus stenting showed that the most commonly employed regimen involved three to five days of aspirin and clopidogrel preoperatively, intravenous heparin during the procedure, and aspirin and clopidogrel for three to six months postoperatively. This was then followed by aspirin alone for a year or more. In contrast, some studies employed warfarin for eight weeks, followed by aspirin for six months or longer. In this same systematic review, there were 2 reports of in-stent thrombosis out of a total of 207 patients with a follow-up ranging from 2 to 108 months (26). These findings suggest that dual antiplatelet therapy with aspirin and clopidogrel may be a suitable initial regimen for human Stentrode^TM^ recipients.

The appropriate duration of antithrombotic medications in Stentrode^TM^ recipients is also presently unknown. This duration may be influenced by the rate of stent incorporation into the vascular endothelium. The risk of stent-associated thrombosis is highest when stent struts are exposed to the bloodstream, and decreases as stent struts are endothelialized. In cardiac coronary stents, for example, the risk of delayed in-stent thrombosis falls when greater than 70% of stent struts are covered by endothelium (48), suggesting that this may be a cut-off value for stent safety. Data from phase angle measurements, histological studies and micro-CT imaging of Stentrodes implanted in sheep suggest that within four weeks of implantation, greater than 85% of stent struts become covered in neo-intima, and after 100 days of implantation, nearly all stent struts are covered in endothelium (38, 39). These findings suggest that a course of antiplatelet therapy as short as 3 months may be sufficient for thrombosis prophylaxis. However, as is the current practice in human venous sinus stenting cohorts, longer durations of antiplatelet therapy are favored.

## Lead Safety: Lessons From Implantable Cardiac Devices

Due to limitations in stent design and electrode size, current endovascular electrode arrays are not capable of wireless operation, and are reliant on transmission of signals through a permanent transvenous lead. The long-term safety of this approach is as yet unknown. Possible complications of a transvenous lead include thrombosis, infection and lead failure, each of which could result in significant neurological morbidity. In order to ensure successful and sustained human application, these complications must be minimized.

Whilst transvenous leads have not been previously utilized in the neurological setting, a number of established cardiac applications rely on the long-term presence of leads within veins: Namely, implantable cardiac defibrillators (ICDs) and permanent pacemakers (PPMs), collectively known as cardiac implantable electronic devices (CIEDs). These technologies provide valuable insights into the risks of intravenous wiring (49), which may be translated to the neurological setting in order to guide the development of endovascular neuromodulation leads that are safe for human use.

### Lead Induced Stenosis and Thrombosis

Lead-associated stenosis and thrombosis are amongst the most serious potential complications of a transvenous lead (49). Several studies have examined the incidence and risk factors for these complications in the cardiac setting (32–36). The quoted rates of lead associated stenosis and occlusion in these studies are highly variable, due to significant differences in study methodology. Data from the most robust studies, which were conducted in a prospective fashion and employed both pre- and post-implantation venography to establish a baseline occlusion rate (Table 2), suggest a modest but not insignificant risk of venous stenosis and occlusion secondary to lead implantation.

**Table 2:** Venous stenosis and occlusion rates following transvenous pacemaker or defibrillator lead insertion.

**References**	**Number of patients and follow-up duration**	**Definition of stenosis**	**Baseline stenosis/occlusion rate**	**New stenosis/occlusion rate**
Oginosawa et al. (33)	Total 131 patients. Baseline DSV performed in all. Post implantation DSV performed in 79 patients, mean follow-up 44 ± 6 months	Venous narrowing > 60% of the maximal diameter of the vessel, measured distal to the stenosis site	Stenosis: 6.8% Occlusion: 6.8%	Stenosis: 13.2% Occlusion: 6.2%
Abu-El-Haija et al. (32)	Total 150 patients. Baseline DSV performed in all. Post implantation DSV performed in 136 patients at 6 months	Maximum (D_max_) and minimum (D_min_) diameters were measured. Stenosis defined as D_min_/D_max_ < 0.4 *and* D_min_ less than 5^th^ centile of all D_max_	Stenosis: 5% Occlusion: 0%	Stenosis: 10% Occlusion 3.6%

Clinical symptoms from lead-associated venous occlusion are exceedingly rare, due to the indolent nature of stenosis progression. (40) Stenosis is driven by two interrelated pathogenic mechanisms: endothelial trauma, which results in fibrous tissue proliferation, and coagulation cascade activation, resulting in thrombus formation. Both are sufficiently gradual to allow for the development of collateral draining vessels to maintain venous return. (40) When symptoms do occur, they typically include upper limb oedema, cyanosis and pain. (49)

Whilst there are several factors that determine the risk of venous stenosis (49), a central question concerning lead design for neurological applications is the role of lead diameter. Although smaller diameter leads may theoretically reduce stenosis risk, the clinical data are equivocal. In adult populations, most implanted cardiac leads range in diameter from 5 to 12 F (1.7 to 4 mm). By comparison, the mean diameter of the innominate vein in adults is 13 mm (50). Whilst multiple leads within the same vein may increase risk of venous occlusion (34), the majority of adult cohort studies demonstrate no significant correlation between individual lead diameter and stenosis risk (33–35).

In contrast, there is some evidence that reducing lead diameters in pediatric patients, who have smaller caliber veins, may confer a lower stenosis risk. Bharmanee et al. compared a low profile 4.1 F (1.4 mm) pacing lead (Medtronic 3830 SelectSecure) with standard 5-7 F leads in a cohort of young patients. Venous stenosis, defined as >60% reduction in diameter, was lower in the SelectSecure group (11%) compared to the group with standard leads (24%) (*P* = 0.0004) (51). Additionally, Figa et al. indexed the cross-sectional area of implanted leads to total body surface area in a cohort of pediatric patients, demonstrating that patients who developed venous obstruction had a higher mean index than patients without obstruction (P < 0.0002) (52). These findings may be more relevant to the neurological setting, given that intracranial veins have a smaller diameter than cardiac veins (53). However, further studies are required to establish the tolerable range of lead to vein ratios that minimize thrombosis risk.

Lead diameter is not the sole determinant of stenosis risk. Some studies have reported that stenosis is more frequently observed at the innominate vein or innominate/SVC junction rather than the subclavian vein, even though the subclavian vein has a smaller diameter (33). This may be due to the proclivity for thrombosis to form where leads are adjacent to the vessel wall, or where there are irregularities in leads, such as tines or defibrillator coils. (35) Furthermore, some literature suggests that patients with previously implanted transvenous pacing leads, whether temporary or permanent, have a higher risk of venous occlusion compared to patients who have not had any prior venous instrumentation. (34, 36, 54) Similarly, venous occlusion appears to correlate with longer procedure duration. (32) These findings support the concept that venous occlusion is driven by endothelial damage.

A number of methods to reduce lead-associated thrombosis have been investigated. Therapeutic anticoagulation may have some protective benefit. In one randomized controlled trial (RCT), (55) which involved 101 CIED recipients at high risk of venous occlusion, defined as having a left ventricular ejection fraction of <40% or having previous temporary transvenous pacing, warfarin therapy (target INR 2.0–3.5) reduced the risk of venous obstruction compared to placebo (risk ratio 0.63, *P* = 0.018). In contrast, the majority of retrospective cohort studies do not show an association between the use of anticoagulation or antiplatelet therapy and reduced stenosis rates (32, 34, 56).

Some investigators have compared the thrombogenicity of different lead coatings. Overall, there is no difference in thrombotic risk associated with two main insulation materials employed in lead design: silicone and polyurethane. (34, 36, 56) More recently, expanded polytetrafluoroethylene (ePTFE), which is highly inert and low in friction, has been used to insulate ICD leads, resulting in lower rates of fibrotic ingrowth and therefore improved ease of lead extraction, (57, 58). However, there is limited data on the rates of vein thrombosis and stenosis related to ePTFE coated leads.

### Infection

Infection remains one of the most feared complications of permanently implanted endovascular devices. In the cerebrovascular system, infection could result in meningitis, septic emboli, and subsequent ischaemic complications. Device associated infection has been comprehensively studied in the cardiac setting, providing parallels that can be translated to neurological applications. However, the epidemiology of CIED-related infections is complex, and must be carefully assessed to determine how this complication may be prevented or managed in the neurological setting.

Most contemporary studies with long-term follow-up report the incidence of CIED-related infection at 1–2% (59–63). Sixty percent of infections occur in the first year post-implantation, and are thought to be related to local contamination of the device at time of implantation. (64, 65) Infections occurring after one year are more likely to be due to blood-borne bacterial seeding. (66) The majority of infections are caused by skin-dwelling *Staphylococcus spp*. (63) CIED-related infections have several different presentations: They can occur as localized pocket infections, infective endocarditis, or bacteraemia. (63) Infective endocarditis constitutes about 20% of total device-related infections. (67) Whilst localized infections can be treated with a course of oral antibiotics, endocarditis, and bacteraemia necessitate total removal of the CIED and intravenous antibiotics. (68) This epidemiology demonstrates that device-related infection has a spectrum of severity. If extrapolated to the neurological setting, this suggests that there may be a considerable buffer between a simply managed, localized infection and a severe complication such as bacterial meningitis.

Several risk factors, modifiable and non-modifiable, increase the rates of CIED-related infection. Patient factors include comorbidities such as diabetes mellitus, end stage renal failure and heart failure. (63) The use of anticoagulation has been shown to increase infection rates by one to three-fold. (65, 69) This may be secondary to an increased risk of bleeding and subsequent pocket haematoma, which provides a fertile environment for bacterial replication. (63) Procedural factors have also been implicated. Longer procedure duration, inexperienced operators and use of temporary pacing prior to lead implantation all increase infection rates. (59, 63) The complexity of the implanted device has a major effect on the likelihood of infection. (63) ICDs, for example, which are larger devices with larger leads, can have infection rates up to five-fold greater than PPMs. (70) This is likely due to an interplay between increased procedure times, the need for larger incisions, implantation pockets with more skin tension and potential dead space, and the presence of greater comorbidities in recipients. (63) Larger leads may also provide increased surface area for bacterial colonization (71). However, the relationship between lead diameter and infection rates in CIEDs remains to be formally investigated.

There are established approaches to infection prevention in CIEDs, that may be successfully translated to the Stentrode^TM^ implantation procedure. Periprocedural intravenous antibiotics provide the mainstay of infection prophylaxis, reducing infection risk by ~75%. (64) Whilst protocols vary, typically an anti-staphylococcal beta-lactam such as flucloxacillin or cephalosporins, are commenced one to two hours before implantation, and continued for several hours or days post-implantation. (64) Given the correlation between device complexity and infection risk, it also stands to reason that the Stentrode^TM^ lead and receiver size should be miniaturized as much as possible without compromising function and durability, and that the insertion technique should be optimized to minimize procedural duration. Although never formally tested in the cardiac setting, there may be rationale to develop antibiotic impregnated leads, given the presence of controlled trials that have shown reduced infection rates with antibiotic coated catheters in different settings, such as central venous catheters, haemodialysis catheters and ventricular drains. (72) If this approach were to be employed in the Stentrode^TM^, the effects of antibiotic impregnation on signal transmission through the lead, and the structural integrity of the Stentrode^TM^ attachment to the lead, would require further study in animal models.

### Lead Failure

Intravenous cardiac leads are subject to a chemically and mechanically stressful environment. (73) Current leads are optimized to withstand these stresses whilst enabling reliable transmission of electrical signals and have an average life expectancy of 10 years. (74) Despite this, some lead models have suffered early mechanical failure, providing significant lessons for future neuro-endovascular lead design.

Key lessons about conductor design can be drawn from the small diameter (6.6 F) Sprint Fidelis defibrillation lead (Medtronic). Originally developed on the presumption that smaller diameter leads have lower thrombosis risk, it has a ten year failure rate of ~20%. (74) In the majority of cases failure is due to conductor fracture. (74) The tendency to fracture is related to its extreme flexibility secondary to small diameter, combined with excessive titanium inclusion bodies within the MP35N conductor alloy (Fort Wayne Metals, Fort Wayne, IN). (74) Fracture risk is further increased when the lead implantation point is in the subclavian or axillary vein rather than the cephalic vein, which subjects the lead to higher bending stress at the costoclavicular junction (75).

There are several possible countermeasures against conductor fractures. The first is to minimize bending stress, by keeping the lead course as anatomically straight as possible, avoiding implantation points that are close to bony junctions and avoiding excess redundant lead. (73) Secondly, improvements in metallic alloys may also reduce fracture rates. For example, the new MP35N LT alloy, with fewer titanium inclusion bodies than its precursor, has greater tensile strength, fatigue resistance and corrosion resistance, and is now the industry standard for lead construction. (73) Lastly, given the aforementioned weak relationship between lead diameter and thrombosis (33–35), excessive reduction in lead diameter should ideally be avoided.

An additional cause of lead failure is insulation abrasion. This occurs either externally (“outside-in”), resulting in electrical failure, or internally (“inside-out”), resulting in externalization of conductors without necessarily impairing electrical function. (74) Typically, leads are designed with a durable external polymer coating such as polyurethane, and a flexible inner coating such as silicone (74). Both components appear to be crucial for the long-term durability of leads. For example, the Riata^TM^ lead (St Jude Medical), which is composed of bare silocone and lacks an external polyurethane layer, has a cable externalization rate of 23.1% and an electrical failure rate of 6.3% (76). To avoid similar negative outcomes in endovascular neuromodulation leads, close attention must be paid to the constituents of the lead coating.

## Discussion and Future Directions

The intracranial venous system represents a promising conduit for neuromodulation devices. The ideal endovascular neuromodulation device would have low thrombogenicity, high biocompatibility without compromising durability, and carry a low infection risk. To achieve this goal, there are several areas in which further research is necessary.

A number of further optimisations of the Stentrode^TM^ array are possible. The current Stentrode^TM^ is essentially a modified self-expanding arterial stent (Solitaire, Covidien) composed of nitinol, with chemically bonded electrodes (14). The original application of the Solitaire stent was for endovascular clot retrieval in the setting of ischaemic stroke (77). It is not necessarily optimized for long-term deployment within the venous sinuses or cerebral veins. Whilst previous human studies of intracranial venous sinus stenting have also successfully used arterial stents (24–31, 42–47), most of these studies are in the setting of stenosed sinuses, where self-expanding stents with high radial forces are justified because they provide intramural pressure to counteract the stenosis. Normal non-stenotic veins would have lower transmural pressures and therefore a stent with higher radial forces may not be necessary. In addition to radial forces, another key variable in stent design is the biocompatibility of the stent material, which may influence in-stent stenosis or thrombosis rates. Novel coatings, such as the Shield^TM^ phosphorylcholine coating utilized on pipeline embolization devices (78), may reduce thrombogenicity and in-stent stenosis, however animal testing and human trials would be required to affirm their efficacy in the Stentrode^TM^ setting.

Given the lack of previous experience with intravenous leads in the brain, lead-associated complications of the Stentrode^TM^ are perhaps of greatest concern. In order to avoid these complications, several factors must be addressed. One of these is lead diameter. Cardiac studies demonstrate an equivocal relationship between lead diameter and thrombosis risk. (33–35, 51, 52) Additionally, there may be limited rationale to reduce lead diameter given the associated increased risk of lead fracture. (74) Higher quality quantitative data are therefore needed to establish the optimal lead: vein diameter ratio without compromising durability or information carrying capacity of the lead, as the present literature does not adequately address this issue. Another key factor is the unique anatomical course of the Stentrode^TM^ lead. The lead may encounter compressive forces at the jugular foramen or be subject to increased bending stress in the neck due to repetitive neck motion. Indeed, in Oxley's study, this issue caused lead fractures in the neck region in 3 sheep. (14) The optimum design modifications to prevent fatigue fracture at these vulnerable sites require further research.

Anatomical variations in cerebral venous drainage patterns must also be considered during patient selection for the Stentrode^TM^ implantation procedure. For example, the Stentrode^TM^ lead must traverse the ostium of the superior anastomotic vein of trolard (VOT), and may therefore theoretically occlude the VOT. This may pose a particularly high risk of territorial cortical venous infarction if there is a lack of sufficient collateral drainage from adjacent bridging veins on the same hemisphere. Furthermore, given that the VOT displays unilateral dominance in up to 50% of cases (79), occlusion of this vein may have serious implications for overall cerebral venous blood flow and intracranial pressure if there is an absence of a co-dominant drainage system on the opposite side. An additional area of concern are the transverse and sigmoid sinuses, which may also display unilateral dominance, and must be traversed by the Stentrode^TM^ lead en route to the internal jugular vein. Occlusion of the transverse sinus on the left side may result in an aphasia due to its drainage of the temporal lobe (80). Furthermore, in patients where there is a significant difference in transverse sinus caliber, occlusion of the dominant sinus may have catastrophic sequelae. Appropriate patient selection using pre-operative cerebral venograms is necessary to minimize the risk of these complications.

Endovascular neuromodulation is a promising field, with numerous potential diagnostic and therapeutic applications. However, significant hurdles must be crossed before this technology reaches routine clinical practice. Robust preventative strategies must be devised in order to minimize complications such as stent- or lead-associated venous thrombosis, device-associated infection, and hardware failure. To optimize the Stentrode^TM^ for future clinical usage, further animal studies and human trials of the device are ultimately necessary. Indeed, the ongoing SWITCH trial, which will test the feasibility and safety of this device in 5 recipients over a follow-up period of 12 months, will provide highly anticipated human data to guide this optimisation process.

## Author Contributions

SR, TO, and NO conjointly conceptualized the idea for the review. SR performed the literature search, analyzed cited studies, and wrote the article. He is guarantor. NO, PM, RS, AM, and TO critically revised the work and made changes and additions to its intellectual content. The corresponding author attests that all listed authors meet authorship criteria and that no others meeting the criteria have been omitted.

## Conflict of Interest

TO, NO, and RS hold stock in Synchron, Inc. Patent application US20140288667A1 filed by TO applies to the Stentrode^TM^. The remaining authors declare that the research was conducted in the absence of any commercial or financial relationships that could be construed as a potential conflict of interest.

## References

[1] Nagahama Y Schmitt AJ Nakagawa D Vesole AS Kamm J Kovach CK Intracranial eEG for seizure focus localization: evolving techniques, outcomes, complications, and utility of combining surface and depth electrodes. J Neurosurg. (2018) 2018:1–13. 10.3171/2018.1.JNS171808 29799342

[2] Cook MJ O'Brien TJ Berkovic SF Murphy M Morokoff A Fabinyi G. Prediction of seizure likelihood with a long-term, implanted seizure advisory system in patients with drug-resistant epilepsy: a first-in-man study. Lancet Neurol. (2013) 12:563–71. 10.1016/S1474-4422(13)70075-9 23642342

[3] Deuschl G Schade-Brittinger C Krack P Volkmann J Schäfer H Bötzel K. A randomized trial of deep-brain stimulation for parkinson's disease. N Engl J Med. (2006) 355:896–908. 10.1056/NEJMoa060281 16943402

[4] Vansteensel MJ Pels EGM Bleichner MG Branco MP Denison T Freudenburg ZV. Fully implanted brain-computer interface in a locked-in patient with aLS. N Engl J Med. (2016) 375:2060–6. 10.1056/NEJMoa1608085 27959736 PMC5326682

[5] Anumanchipalli GK Chartier J Chang EF. Speech synthesis from neural decoding of spoken sentences. Nature. (2019) 568:493–8. 10.1038/s41586-019-1119-1 31019317 PMC9714519

[6] Akbari H Khalighinejad B Herrero JL Mehta AD Mesgarani N. Towards reconstructing intelligible speech from the human auditory cortex. Sci Rep. (2019) 9:874. 10.1038/s41598-018-37359-z 30696881 PMC6351601

[7] Collinger JL Wodlinger B Downey JE Wang W Tyler-Kabara EC Weber DJ. High-performance neuroprosthetic control by an individual with tetraplegia. Lancet. (2013) 381:557–64. 10.1016/S0140-6736(12)61816-9 23253623 PMC3641862

[8] Hamer HM Morris HH Mascha EJ Karafa MT Bingaman WE Bej MD. Complications of invasive video-EEG monitoring with subdural grid electrodes. Neurology. (2002) 58:97–103. 10.1212/WNL.58.1.97 11781412

[9] Ben-Haim S Asaad WF Gale JT Eskandar EN. Risk factors for hemorrhage during microelectrode-guided deep brain stimulation and the introduction of an improved microelectrode design. Neurosurgery. (2009) 64:754–62; discussion 62–3. 10.1227/01.NEU.0000339173.77240.34 19349834

[10] Cardinale F Cossu M Castana L Casaceli G Schiariti MP Miserocchi A. Stereoelectroencephalography: surgical methodology, safety, and stereotactic application accuracy in 500 procedures. Neurosurgery. (2013) 72:353–66; discussion 66. 10.1227/NEU.0b013e31827d1161 23168681

[11] Boviatsis EJ Stavrinou LC Themistocleous M Kouyialis AT Sakas DE. Surgical and hardware complications of deep brain stimulation. A seven-year experience and review of the literature. Acta Neurochir (Wien). (2010) 152:2053–62. 10.1007/s00701-010-0749-8 20658301

[12] Polikov VS Tresco PA Reichert WM. Response of brain tissue to chronically implanted neural electrodes. J Neurosci Methods. (2005) 148:1–18. 10.1016/j.jneumeth.2005.08.015 16198003

[13] Satzer D Lanctin D Eberly LE Abosch A. Variation in deep brain stimulation electrode impedance over years following electrode implantation. Stereotact Funct Neurosurg. (2014) 92:94–102. 10.1159/000358014 24503709 PMC4531050

[14] Oxley TJ Opie NL John SE Rind GS Ronayne SM Wheeler TL. Minimally invasive endovascular stent-electrode array for high-fidelity, chronic recordings of cortical neural activity. Nat Biotechnol. (2016) 34:320–7. 10.1038/nbt.3428 26854476

[15] Opie NL John SE Rind GS Ronayne SM Wong YT Gerboni G. Focal stimulation of the sheep motor cortex with a chronically implanted minimally invasive electrode array mounted on an endovascular stent. Nat Biomed Eng. (2018) 2:907–14. 10.1038/s41551-018-0321-z 31015727

[16] Teplitzky BA Connolly AT Bajwa JA Johnson MD. Computational modeling of an endovascular approach to deep brain stimulation. J Neural Eng. (2014) 11:026011. 10.1088/1741-2560/11/2/026011 24608363

[17] Sefcik RK Opie NL John SE Kellner CP Mocco J Oxley TJ. The evolution of endovascular electroencephalography: historical perspective and future applications. Neurosurg Focus. (2016) 40:E7. 10.3171/2016.3.FOCUS15635 27132528

[18] Penn RD Hilal SK Michelsen WJ Goldensohn ES Driller J. Intravascular intracranial eEG recording. Technical note. J Neurosurg. (1973) 38:239–43. 10.3171/jns.1973.38.2.0239 4632831

[19] Mikuni N Ikeda A Terada K Taki W Kikuchi H Kimura J. Frontopolar ictal epileptiform discharges on scalp electroencephalogram in temporal lobe epilepsy. J Clin Neurophysiol. (1997) 14:507–12. 10.1097/00004691-199711000-00007 9458057

[20] Kunieda T Ikeda A Mikuni N Ohara S Sadato A Taki W. Use of cavernous sinus eEG in the detection of seizure onset and spread in mesial temporal lobe epilepsy. Epilepsia. (2000) 41:1411–9. 10.1111/j.1528-1157.2000.tb00116.x 11077454

[21] Thömke F Stoeter P Stader D. Endovascular electroencephalography during an intracarotid amobarbital test with simultaneous recordings from 16 electrodes. J Neurol Neurosurg Psychiatry. (1998) 64:565. 10.1136/jnnp.64.4.565 9576562 PMC2170034

[22] Bower MR Stead M Van Gompel JJ Bower RS Sulc V Asirvatham SJ. Intravenous recording of intracranial, broadband eEG. J Neurosci Methods. (2013) 214:21–6. 10.1016/j.jneumeth.2012.12.027 23313850 PMC3593671

[23] John SE Opie NL Wong YT Rind GS Ronayne SM Gerboni G. Signal quality of simultaneously recorded endovascular, subdural and epidural signals are comparable. Sci Rep. (2018) 8:8427. 10.1038/s41598-018-36257-8 29849104 PMC5976775

[24] Puffer RC Mustafa W Lanzino G. Venous sinus stenting for idiopathic intracranial hypertension: a review of the literature. J Neurointerv Surg. (2013) 5:483–6. 10.1136/neurintsurg-2012-010468 22863980

[25] Lai LT Danesh-Meyer HV Kaye AH. Visual outcomes and headache following interventions for idiopathic intracranial hypertension. J Clin Neurosci. (2014) 21:1670–8. 10.1016/j.jocn.2014.02.025 24974193

[26] Teleb MS Cziep ME Lazzaro MA Gheith A Asif K Remler B. Idiopathic intracranial hypertension. A systematic analysis of transverse sinus stenting. Interv Neurol. (2013) 2:132–43. 10.1159/000357503 24999351 PMC4080637

[27] Satti SR Leishangthem L Chaudry MI. Meta-Analysis of cSF diversion procedures and dural venous sinus stenting in the setting of medically refractory idiopathic intracranial hypertension. AJNR Am J Neuroradiol. (2015) 36:1899–904. 10.3174/ajnr.A4377 26251432 PMC7965019

[28] Starke RM Wang T Ding D Durst CR Crowley RW Chalouhi N. Endovascular treatment of venous sinus stenosis in idiopathic intracranial hypertension: complications, neurological outcomes, and radiographic results. Sci World J. (2015) 2015:140408. 10.1155/2015/140408 26146651 PMC4471318

[29] Nicholson P Brinjikji W Radovanovic I Hilditch CA Tsang ACO Krings T. Venous sinus stenting for idiopathic intracranial hypertension: a systematic review and meta-analysis. J Neurointerv Surg. (2019) 11:380–5. 10.1136/neurintsurg-2018-014172 30166333

[30] Raper DMS Ding D Chen CJ Buell TJ Crowley RW Liu KC. Patency of the vein of labbé after venous stenting of the transverse and sigmoid sinuses. J Neurointerv Surg. (2017) 9:587–90. 10.1136/neurintsurg-2016-012903 28073989

[31] Levitt MR Albuquerque FC Ducruet AF Kalani MY Mulholland CB McDougall CG. Venous sinus stenting for idiopathic intracranial hypertension is not associated with cortical venous occlusion. J Neurointerv Surg. (2016) 8:594–5. 10.1136/neurintsurg-2015-011692 25854688

[32] Korkeila P Nyman K Ylitalo A Koistinen J Karjalainen P Lund J. Venous obstruction after pacemaker implantation. Pacing Clin Electrophysiol. (2007) 30:199–206. 10.1111/j.1540-8159.2007.00650.x 17338716

[33] Oginosawa Y Abe H Nakashima Y. The incidence and risk factors for venous obstruction after implantation of transvenous pacing leads. Pacing Clin Electrophysiol. (2002) 25:1605–11. 10.1046/j.1460-9592.2002.01605.x 12494619

[34] Abu-El-Haija B Bhave PD Campbell DN Mazur A Hodgson-Zingman DM Cotarlan V. Venous stenosis after transvenous lead placement: a Study of outcomes and risk factors in 212 consecutive patients. J Am Heart Assoc. (2015) 4:e001878. 10.1161/JAHA.115.001878 26231843 PMC4599456

[35] Sticherling C Chough SP Baker RL Wasmer K Oral H Tada H. Prevalence of central venous occlusion in patients with chronic defibrillator leads. Am Heart J. (2001) 141:813–6. 10.1067/mhj.2001.114195 11320371

[36] Lickfett L Bitzen A Arepally A Nasir K Wolpert C Jeong KM. Incidence of venous obstruction following insertion of an implantable cardioverter defibrillator. A study of systematic contrast venography on patients presenting for their first elective ICD generator replacement. Europace. (2004) 6:25–31. 10.1016/j.eupc.2003.09.001 14697723

[37] Oxley TJ Opie NL Rind GS Liyanage K John SE Ronayne S. An ovine model of cerebral catheter venography for implantation of an endovascular neural interface. J Neurosurg. (2018) 128:1020–7. 10.3171/2016.11.JNS161754 28452616

[38] Opie NL Rind GS John SE Ronayne SM Grayden DB Burkitt AN. Feasibility of a chronic, minimally invasive endovascular neural interface. Conf Proc IEEE Eng Med Biol Soc. (2016) 2016:4455–8. 10.1109/EMBC.2016.7591716 28269267

[39] Opie NL van der Nagel NR John SE Vessey K Rind GS Ronayne SM. Micro-CT and histological evaluation of an neural interface implanted within a blood vessel. IEEE Trans Biomed Eng. (2017) 64:928–34. 10.1109/TBME.2016.2552226 27337706

[40] Stoney WS Addlestone RB Alford WC Burrus GR Frist RA Thomas CS. The incidence of venous thrombosis following long-term transvenous pacing. Ann Thorac Surg. (1976) 22:166–70. 10.1016/S0003-4975(10)63980-X 788660

[41] Sheth SA Trieu H Liebeskind DS Saver JL Szeder V Jahan R. Venous collateral drainage patterns predict clinical worsening in dural venous sinus thrombosis. J Neurointerv Surg. (2018) 10:171–5. 10.1136/neurintsurg-2016-012941 28265010

[42] Raper DMS Buell TJ Ding D Pomeraniec IJ Crowley RW Liu KC. A pilot study and novel angiographic classification for superior sagittal sinus stenting in patients with non-thrombotic intracranial venous occlusive disease. J Neurointerv Surg. (2018) 10:74–7. 10.1136/neurintsurg-2016-012906 28082447

[43] Matsumoto N Kuramoto Y Shinoda N Ueno Y. A case of stenting for acute cerebral venous sinus thrombosis in the superior sagittal sinus. Interv Neuroradiol. (2016) 22:709–10. 10.1177/1591019916663093 27535922 PMC5564362

[44] Adachi H Mineharu Y Ishikawa T Imamura H Yamamoto S Todo K. Stenting for acute cerebral venous sinus thrombosis in the superior sagittal sinus. Interv Neuroradiol. (2015) 21:719–23. 10.1177/1591019915609120 26494402 PMC4757359

[45] Ohara N Toyota S Kobayashi M Wakayama A. Superior sagittal sinus dural arteriovenous fistulas treated by stent placement for an occluded sinus and transarterial embolization. A case report. Interv Neuroradiol. (2012) 18:333–40. 10.1177/159101991201800314 22958774 PMC3442309

[46] Entezami P Gooch MR Dalfino J. Endovascular stenting of the superior sagittal sinus to alleviate venous compression caused by a parasagittal meningioma. BMJ Case Rep. (2019) 12:4. 10.1136/bcr-2018-227935 30954959 PMC6453347

[47] Ganesan D Higgins JN Harrower T Burnet NG Sarkies NJ Manford M. Stent placement for management of a small parasagittal meningioma. Technical note. J Neurosurg. (2008) 108:377–81. 10.3171/JNS/2008/108/2/0377 18240939

[48] Finn AV Joner M Nakazawa G Kolodgie F Newell J John MC. Pathological correlates of late drug-eluting stent thrombosis: strut coverage as a marker of endothelialization. Circulation. (2007) 115:2435–41. 10.1161/CIRCULATIONAHA.107.693739 17438147

[49] Donnelly J Gabriels J Galmer A Willner J Beldner S Epstein LM. Venous obstruction in cardiac rhythm device therapy. Curr Treat Options Cardiovasc Med. (2018) 20:64. 10.1007/s11936-018-0664-5 29995225

[50] Sanjeev S Karpawich PP. Superior vena cava and innominate vein dimensions in growing children : an aid for interventional devices and transvenous leads. Pediatr Cardiol. (2006) 27:414–9. 10.1007/s00246-006-1133-6 16830087

[51] Bharmanee A Zelin K Sanil Y Gupta P Karpawich PP. Comparative chronic valve and venous effects of lumenless versus stylet-Delivered pacing leads in patients with and without congenital heart. Pacing Clin Electrophysiol. (2015) 38:1343–50. 10.1111/pace.12728 26256093

[52] Figa FH McCrindle BW Bigras JL Hamilton RM Gow RM. Risk factors for venous obstruction in children with transvenous pacing leads. Pacing Clin Electrophysiol. (1997) 20(8 Pt 1):1902–9. 10.1111/j.1540-8159.1997.tb03594.x 9272526

[53] Andrews BT Dujovny M Mirchandani HG Ausman JI. Microsurgical anatomy of the venous drainage into the superior sagittal sinus. Neurosurgery. (1989) 24:514–20. 10.1227/00006123-198904000-00005 2710296

[54] Da Costa SS Scalabrini Neto A Costa R Caldas JG Martinelli Filho M. Incidence and risk factors of upper extremity deep vein lesions after permanent transvenous pacemaker implant: a 6-month follow-up prospective study. Pacing Clin Electrophysiol. (2002) 25:1301–6. 10.1046/j.1460-9592.2002.01301.x 12380764

[55] Costa R Da Silva KR Rached R Martinelli Filho M Carnevale FC Moreira LF Prevention of venous thrombosis by warfarin after permanent transvenous leads implantation in high-risk patients. Pacing Clin Electrophysiol. (2009) 32 Suppl 1:S247–51. 10.1111/j.1540-8159.2008.02295.x 19250106

[56] Goto Y Abe T Sekine S Sakurada T. Long-term thrombosis after transvenous permanent pacemaker implantation. Pacing Clin Electrophysiol. (1998) 21:1192–5. 10.1111/j.1540-8159.1998.tb00177.x 9633060

[57] Di Cori A Bongiorni MG Zucchelli G Segreti L Viani S Paperini L. Transvenous extraction performance of expanded polytetrafluoroethylene covered iCD leads in comparison to traditional iCD leads in humans. Pacing Clin Electrophysiol. (2010) 33:1376–81. 10.1111/j.1540-8159.2010.02879.x 20735715

[58] Wilkoff BL Belott PH Love CJ Scheiner A Westlund R Rippy M. Improved extraction of ePTFE and medical adhesive modified defibrillation leads from the coronary sinus and great cardiac vein. Pacing Clin Electrophysiol. (2005) 28:205–11. 10.1111/j.1540-8159.2005.40029.x 15733180

[59] Klug D Balde M Pavin D Hidden-Lucet F Clementy J Sadoul N. Risk factors related to infections of implanted pacemakers and cardioverter-defibrillators: results of a large prospective study. Circulation. (2007) 116:1349–55. 10.1161/CIRCULATIONAHA.106.678664 17724263

[60] Rahman R Saba S Bazaz R Gupta V Pokrywka M Shutt K. Infection and readmission rate of cardiac implantable electronic device insertions: an observational single center study. Am J Infect Control. (2016) 44:278–82. 10.1016/j.ajic.2015.10.006 26704827

[61] Polyzos KA Konstantelias AA Falagas ME. Risk factors for cardiac implantable electronic device infection: a systematic review and meta-analysis. Europace. (2015) 17:767–77. 10.1093/europace/euv053 25926473

[62] Prutkin JM Reynolds MR Bao H Curtis JP Al-Khatib SM Aggarwal S. Rates of and factors associated with infection in 200 909 medicare implantable cardioverter-defibrillator implants: results from the national cardiovascular data registry. Circulation. (2014) 130:1037–43. 10.1161/CIRCULATIONAHA.114.009081 25081281

[63] Arnold CJ Chu VH. Cardiovascular implantable electronic device infections. Infect Dis Clin North Am. (2018) 32:811–25. 10.1016/j.idc.2018.06.004 30241711

[64] Da Costa A Lelièvre H Kirkorian G Célard M Chevalier P Vandenesch F. Role of the preaxillary flora in pacemaker infections: a prospective study. Circulation. (1998) 97:1791–5. 10.1161/01.CIR.97.18.1791 9603533

[65] Özcan C Raunsø J Lamberts M Køber L Lindhardt TB Bruun NE. Infective endocarditis and risk of death after cardiac implantable electronic device implantation: a nationwide cohort study. Europace. (2017) 19:1007–14. 10.1093/europace/euw404 28073883

[66] Korantzopoulos P Sideris S Dilaveris P Gatzoulis K Goudevenos JA. Infection control in implantation of cardiac implantable electronic devices: current evidence, controversial points, and unresolved issues. Europace. (2016) 18:473–8. 10.1093/europace/euv260 26516219

[67] Le KY Sohail MR Friedman PA Uslan DZ Cha SS Hayes DL. Clinical predictors of cardiovascular implantable electronic device-related infective endocarditis. Pacing Clin Electrophysiol. (2011) 34:450–9. 10.1111/j.1540-8159.2010.02991.x 21208230

[68] Baddour LM Epstein AE Erickson CC Knight BP Levison ME Lockhart PB. Update on cardiovascular implantable electronic device infections and their management: a scientific statement from the american heart association. Circulation. (2010) 121:458–77. 10.1161/CIRCULATIONAHA.109.192665 20048212

[69] Lekkerkerker JC van Nieuwkoop C Trines SA van der Bom JG Bernards A van de Velde ET. Risk factors and time delay associated with cardiac device infections: leiden device registry. Heart. (2009) 95:715–20. 10.1136/hrt.2008.151985 19036758

[70] Romeyer-Bouchard C Da Costa A Dauphinot V Messier M Bisch L Samuel B. Prevalence and risk factors related to infections of cardiac resynchronization therapy devices. Eur Heart J. (2010) 31:203–10. 10.1093/eurheartj/ehp421 19875388

[71] Lloyd DP Allen RJ. Competition for space during bacterial colonization of a surface. J R Soc Interface. (2015) 12:0608. 10.1098/rsif.2015.0608 26333814 PMC4614474

[72] Kondo Y Ueda M Kobayashi Y Schwab JO. New horizon for infection prevention technology and implantable device. J Arrhythm. (2016) 32:297–302. 10.1016/j.joa.2016.02.007 27588153 PMC4996843

[73] Lau E Leads and electrodes for cardiac implantable electronic devices. In: Ellenbogen K editor. Clinical Cardiac Pacing, Defibrillation and Resynchronization Therapy, Fifth Edition. 5th ed (Amsterdam: Elsevier) (2018) 10.1016/B978-0-323-37804-8.00011-0

[74] Swerdlow CD Kalahasty G Ellenbogen KA. Implantable cardiac defibrillator lead failure and management. J Am Coll Cardiol. (2016) 67:1358–68. 10.1016/j.jacc.2015.12.067 26988958

[75] Birnie DH Parkash R Exner DV Essebag V Healey JS Verma A. Clinical predictors of fidelis lead failure: report from the canadian heart rhythm society device committee. Circulation. (2012) 125:1217–25. 10.1161/CIRCULATIONAHA.111.053744 22311781

[76] Zeitler EP Pokorney SD Zhou K Lewis RK Greenfield RA Daubert JP. Cable externalization and electrical failure of the riata family of implantable cardioverter-defibrillator leads: a systematic review and meta-analysis. Heart Rhythm. (2015) 12:1233–40. 10.1016/j.hrthm.2015.03.005 25998139

[77] Goyal M Demchuk AM Menon BK Eesa M Rempel JL Thornton J. Randomized assessment of rapid endovascular treatment of ischemic stroke. N Engl J Med. (2015) 372:1019–30. 10.1056/NEJMoa1414905 25671798

[78] Martínez-Galdámez M Lamin SM Lagios KG Liebig T Ciceri EF Chapot R. Treatment of intracranial aneurysms using the pipeline flex embolization device with shield technology: angiographic and safety outcomes at 1-year follow-up. J Neurointerv Surg. (2019) 11:396–9. 10.1136/neurintsurg-2018-014204 30262655 PMC6582709

[79] Kawamata T Matsumoto K Goto N Kohda M Morphometric anatomy of superficial cerebral veins and cerebral sulci. Showa Univ J Med Sci. (1996) 8:103 10.15369/sujms1989.8.103

[80] Bousser MG Ferro JM. Cerebral venous thrombosis: an update. Lancet Neurol. (2007) 6:162–70. 10.1016/S1474-4422(07)70029-7 17239803

